# Single-Molecule Imaging in Living Plant Cells: A Methodological Review

**DOI:** 10.3390/ijms22105071

**Published:** 2021-05-11

**Authors:** Ai-Yu Guo, Ya-Mei Zhang, Liu Wang, Di Bai, Ya-Peng Xu, Wen-Qiang Wu

**Affiliations:** State Key Laboratory of Crop Stress Adaptation and Improvement, Key Laboratory of Plant Stress Biology, School of Life Sciences, Henan University, Kaifeng 475001, China; 104752150070@vip.henu.edu.cn (A.-Y.G.); 104753201557@henu.edu.cn (Y.-M.Z.); 104753191190@henu.edu.cn (L.W.); 104753201530@henu.edu.cn (D.B.); 104753181161@vip.henu.edu.cn (Y.-P.X.)

**Keywords:** single-molecule imaging, plant imaging, light sheet, methodology

## Abstract

Single-molecule imaging is emerging as a revolutionary approach to studying fundamental questions in plants. However, compared with its use in animals, the application of single-molecule imaging in plants is still underexplored. Here, we review the applications, advantages, and challenges of single-molecule fluorescence imaging in plant systems from the perspective of methodology. Firstly, we provide a general overview of single-molecule imaging methods and their principles. Next, we summarize the unprecedented quantitative details that can be obtained using single-molecule techniques compared to bulk assays. Finally, we discuss the main problems encountered at this stage and provide possible solutions.

## 1. Introduction

In traditional ensemble approaches, all molecules of identical types are assumed to be synchronous, but they act stochastically in many cases. As a result, the conventional “averaged” measurements lose many reaction details [1,2]. This is an impediment to uncovering biological molecular mechanisms. Due to the continuous development of technology in recent decades, single-molecule fluorescence technology has made great progress and has evolved into a valuable biophysical research method that enables researchers to observe the real-time behavior of individual biomolecules and has revolutionized our ability to truly understand their detailed characteristics [3,4,5,6,7,8]. Investigating biomolecules using single-molecule techniques is the natural extension and is undoubtedly inevitable in living plant cells. Here, we provide a general overview of the single-molecule imaging methods used in living plants from the perspective of methodology, considering that many published comprehensive reviews have enumerated the biological applications [9,10]. It should be emphasized that the single-molecule referred to here is not single-particle, such as vesicles. We believe that this review may be helpful in understanding the frontier of single-molecule imaging in living plant cells.

## 2. Brief History of Single-Molecule Imaging

In 1961, Boris Rotman first proposed the possibility of using fluorescence microscopy to infer the presence of single molecules [11]. In 1976, Thomas Hirshfeld realized the direct detection of the protein globulin labeled with ≈100 fluorescein molecules [12]. The detection of single dye molecules was achieved by Michel Orrit and Jacky Bernard at cryogenic temperatures in 1990 [13]. However, most organisms live in non-cryogenic environments. The single-molecule detection of fluorescent molecules in the liquid phase at room temperature was realized in 1994 [14]. Sako then performed the first single-molecule measurements in living cells in 2000 [15]. Due to the poor optical properties of plant cells, it was not until 2011 that Lin’s laboratory applied single-molecule fluorescence technology to plant cells for the first time [16]. Since then, single-molecule imaging has been applied to many complex biological processes [9,17,18,19].

## 3. Single-Molecule Labeling and Imaging Strategies

When one protein is studied by single-molecule imaging, it is labeled with a fluorophore so that it can be distinguished from the background. Compared with imaging in animals, plant imaging is mainly limited by the large amount of autofluorescence and thick cell walls. Plant autofluorescence arises from a variety of compounds, such as chlorophyll and lignin, and has been used for imaging and diagnosis [20,21]. However, autofluorescence results in poor imaging backgrounds and interferes with the detection of single-molecule signals. Considering the poor imaging environment in plants, in order to obtain a high signal-to-noise ratio in living cells, two basic principles need to be considered: first, the fluorophore should emit more photons than the competing spots nearby, and second, to reduce the competition, the excited object of interest or volume should be as small as possible.

### 3.1. Fluorophores Used for Single-Molecule Research

Fluorescent molecules used in imaging can be divided into three categories: fluorescent proteins, small-molecule fluorescent dyes, and luminescent nanoparticles.

#### 3.1.1. Fluorescent Proteins

Because fluorescent proteins are genetically encoded proteins, the one-to-one labeling of a target protein and a fluorophore can be achieved through gene fusion [22]. This process is very well suited for in vivo applications. Wild-type green fluorescent protein (GFP) was isolated from the jellyfish *Aequorea victoria* in 1962. Since then, in order to optimize its physical optical properties, many different mutants have been engineered [23,24]. Among them, two mutants with improved fluorescence brightness are S65T and EGFP (F64L/S65T) [25]. EGFP has been widely used in confocal fluorescence imaging experiments. Due to electrostatic adsorption, GFP tends to form a homodimer, which may limit its application for understanding the oligomerization of target proteins [26]. Fortunately, the mutations of interface hydrophobic residues (A206, L221, or F223) with positively charged residues are able to observably reduce the dimerization [26], and the mutant A206K has been successfully used in plant cells [22]. Red fluorescent protein was first extracted from marine organisms. Typical examples include DsRed from *Discosoma* [27] and HcRed from *Heteractis crispa* [28]. Most of the wild-type red fluorescent proteins obtained in the early stage are tetrameric and are often toxic or disruptive [27,29]. mRFP1 was the first true monomer, obtained from DsRed. However, its application in vivo may affect the function of the target protein [30]. After continuous improvement, the commonly used red fluorescent mutants are mCherry and TagRFP, and new mutants with better spectral properties are being developed all the time [30,31,32].

There are many other colors of fluorescent proteins and their variants, and fluorescent protein modification is still a frontier research field. However, at present, only GFP and mCherry are widely used as co-localization partners in plant single-molecule experiments [33]. Fluorescence intensity is affected by environmental conditions [34,35], and the fluorescent proteins used in animals may not be suitable for plants. Therefore, their use needs to be verified in plants. While fluorescent proteins are the most widely used fluorescent molecules in living cells, they have disadvantages, including their large size (≈27 kDa), which may affect the behaviors of the targets. In addition, the light stability and brightness of fluorescent proteins are much lower than those of chemical small dyes and luminescent nanoparticles.

In addition to enhancing the emission intensity of a single fluorescent protein, increasing the number of fluorescent proteins on a single molecule can also improve the signal-to-noise ratio. This method was used for the design of tdTomato [31]. However, more repetitive tandem fluorescent proteins will encounter problems in vector construction and protein expression. In recent years, Marvin E. Tanenbaum and colleagues successively developed the SunTag [36] system and MoonTag system [37] for signal amplification. In these systems, one type of nanobody fused with a fluorescent protein is used to bind special tandem short peptides (typically 24 copies) fused with a target protein; thus, one protein will be labeled by many fluorescent proteins from antibody–antigen recognition. Using this system together with RNA-protein recognition systems such as the MS2 system [38] and PP7 system [39], researchers studied the transcription and translation processes at the single-molecule level [40,41,42]. Although these amplification systems have been used effectively in plants [43], they may not be suitable for single-molecule tracking, because three tandem fluorescent proteins will affect the movement of protein [44]. On the basis of the principle of fluorescent proteins, many fluorescent RNAs have been developed recently to mimic fluorescent proteins [45], such as spinach [46], broccoli [47], corn [48], and pepper [49]. These fluorescent RNAs provide an opportunity to light up RNAs directly in cells. In addition, some of them have been used in plant systems [50].

#### 3.1.2. Small-Molecule Fluorescent Dyes

This type of fluorescent molecule has been used for decades and is commercially available. The benefits of this type of molecule include its small size, wide spectral range, and high photostability. Small-molecule fluorescent dyes have been widely used in single-molecule experiments in vitro [4,8,51], and their use has realized the labeling of plant and animal organelles [52,53]. However, in terms of amino acid-based protein labeling, the specificity is usually not guaranteed in cells due to competition from other proteins. In recent years, in order to solve the problem of specific labeling, self-labeling protein tags have been developed [54], including SNAP-tag (NEB) [55], CLIP-tag (NEB) [56], and Halo-tag (Promega) [57]. In these systems, the tagged proteins of interest are genetically fused to self-labelling enzyme tags, which are further enzymatically labeled by specific ligands conjugated to different organic dyes. Taking advantage of this system, researchers have widely used chemical fluorescent small molecules in animals and bacteria at the single-molecule level [2]. Although this method combines the advantages of both fluorescent dyes and gene fusion techniques and has been successfully used in plants [58], single-molecule imaging has still not been achieved. Because of the presence of cell walls, fluorescent molecules cannot easily enter cells, and it is not easy to adequately rinse out free fluorescent molecules as it is in animal cells.

#### 3.1.3. Luminescent Nanoparticles

Quantum dots (QDs), the first generation of luminescent nanoparticles, are representative. These fluorophores are characterized by high brightness and resistance to photobleaching, being able to form a variety of different absorption and emission bands by changing their physical morphology [59]. They are commercially available and have been widely used in single-molecule imaging in animal cells [60,61]. QDs have been successfully used to label calmodulin in plant cells [62]. However, the limitations of QDs are also noteworthy. First, compared with traditional organic dyes, QDs tend to blink irregularly [63]. This drawback limits their applications for single-molecule tracking [64]. Although essentially nonblinking QDs have also been developed [65], more research in this area needs to be conducted. Second, QDs are much larger than small-molecule dyes and fluorescent proteins, and therefore they may affect the diffusion rate and pattern of the target [66]. Third, there is currently no effective way for QDs to pass smoothly through plant cell walls. Finally, in terms of specific labeling, QDs have the same problems as the above small-molecule fluorescent dyes.

Other common luminescent nanoparticles include up-conversion nanocrystals (UCNPs), polymer dots (PDots), fluorescent nanodiamonds (FNDs), and carbon-based nanodots (CDots). Their properties and usage in animals at the single-molecule level have been well reviewed [67]. There have been some attempts to apply these nanoparticles in plants [68], but they are still at an early stage, and these nanoparticles have not been used for single-molecule imaging. The absorption, accumulation, and imaging of UCNPs [69,70], PDots [71], FNDs [72], and CDots [73] in plants have been studied. Similar to QDs, their specific labeling is also a major problem. However, because of their excellent brightness, efforts are still being made to implement the wide application of QDs in the single-molecule detection of plant cells.

### 3.2. Instrumentations for Single-Molecule Research

**Figure 1:**
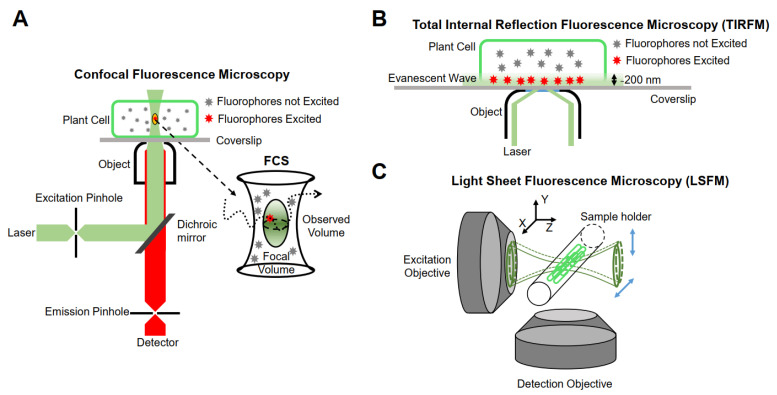
Schematic representation of instrumentations for single-molecule research. (**A**) Confocal fluorescence microscopy. (**B**) Total internal reflection fluorescence microscopy (TIRFM). (**C**) Light sheet fluorescence microscopy (LSFM).

The realization of single-molecule fluorescence detection needs to be based on different research purposes, and excitation modes and detection methods should be designed. Currently, single-molecule imaging methods are mainly based on the following three methods in living plants (Figure 1).

#### 3.2.1. Confocal Fluorescence Microscopy

Minsky proposed the concept of confocal microscopy in 1961 [74]. It uses a diffraction-limited point of light to illuminate the sample, and then all of the fluorescence information emitted is collected by a point detector, which consists of a detector and a front pinhole, removing the majority of light outside of the focal plane. In addition, the data of the whole sample are obtained by means of transverse and axial scanning. Although the emissions from out-of-focus molecules can be filtered, the useless out-of-focus excitation will lead to premature bleaching and phototoxicity. Furthermore, because of the point-scanning acquisition, the imaging speed is relatively slow for fast molecular detection [75]. Therefore, this type of microscope is not suitable for single-molecule detection in living plant cells. Confocal microscopes combined with multiple detectors and relevant analysis systems have partly overcome their limitations. For example, fluorescence correlation spectroscopy (FCS) and fluorescence cross-correlation spectroscopy (FCCS) detect small, defined illumination volume diffusion fluorescence intensity fluctuations of fluorescent molecules and analyze the time-dependence fluctuations using auto-correlation analysis to obtain the fluorescent molecular mobility, diffusion, concentration, and aggregation [76,77].

The first literature on the use of FCS in plants was published in 1999, when the diffusion of a cytosolic GFP mutant S65T was investigated in tobacco, and two-photon excitation was proven to be a better choice to improve signal quality for turbid plant cells [78]. Using FCS/FCCS, the endocytic pathways of RbohD under salt stress were studied [79], and the accumulation of PLDδ-GFP on the membrane under pathogen stimulation was confirmed [80]. FCS/FCCS has gradually become a standard method for plant single-molecule imaging, but it is not a true single-molecule technology because it does not track individual molecules. In addition, it yields an average result, although the imaging volume is small. FCS/FCCS is also not suitable for slow-moving and immobile objects, being inaccurate at high concentrations of fluorescent molecules.

#### 3.2.2. Total Internal Reflection Fluorescence Microscopy (TIRFM)

Conventional fluorescence microscopes use vertical excitation of the sample. As a result, the excitation volume in the *z*-axis direction is very large, leading to a low signal-to-noise ratio in the resulting image. Total internal reflection fluorescence (TIRF) microscopy is currently the most commonly used imaging method in plants, taking advantage of its incomparable signal-to-noise ratio [75]. It uses the evanescent wave generated when the incident light experiences total internal reflection, or a highly inclined and laminated optical sheet (HILO) to light up only a partial volume (for TIRF less than 200 nm) [81] in order to obtain the dynamic behavior of a single fluorescent molecule in time and space [82].

TIRFM was first achieved in plant single-molecule imaging in 2011, when Lin’s group first detected and studied single-molecule PIP2;1 and found that it was distributed heterogeneously [16]. Since then, TIRFM has been widely used to study biological processes occurring on or near cell membranes, such as cell signaling and cytoskeleton assembly [83,84]. However, because of the principle of TIRFM, most studies in living plant cells have been limited to membrane or near-membrane studies.

#### 3.2.3. Light Sheet Fluorescence Microscopy (LSFM)

Although TIRFM technology realizes an ultra-thin excitation surface, its use is limited to two-dimensional imaging. The emergence of LSFM allows for the illumination plane to be oriented in any desired position, thus enabling researchers to achieve high-resolution imaging in three dimensions. The fundamental principle of LSFM is to use two vertical objective lenses: one is the lighting system, and the other is the detection system. The lighting system forms an extremely thin sheet of excitation light, which illuminates only the focal plane of the sample, and then scans the sample layer by layer to obtain three-dimensional images. At present, LSFM has been used for plant imaging at the tissue level [85] and is able to be extended to the single-molecule level [86]. The illumination strategies of LSFM can be categorized into three types: Gaussian light sheet illumination, Bessel beam selective-plane illumination, and lattice light sheet illumination [87]. Compared with Gaussian light sheet illumination, Bessel beam selective-plane illumination and lattice light sheet illumination have thinner and nondiffracting beams, and thus have better backgrounds resulting from out-of-focus molecules and a better signal-to-noise ratio. Gaussian light sheet illumination has been successfully used to detect single PMA4-mGFP in the root hairs of *Arabidopsis* at the single-molecule level [88], and the applications of Bessel beam selective-plane illumination and lattice light sheet illumination in plants are worth investigating.

#### 3.2.4. Super-Resolution and Other Cutting-Edge Single-Molecule Imaging Methods

Thus far, besides limitation to a surface, single-molecule research is also restricted to high spatiotemporal resolution and long-time tracking. For long-time tracking, recently, 3D-SMART (3D single-molecule active real-time tracking method) was presented [89]. When this active feedback tracking strategy is used, single-molecule biomacromolecules can be directly monitored with a duration of about 16 s (step response ≈ 0.1 ms), and tracking rates can be up to 10 µm^2^/s. For more precise positioning or achieving single-molecule detection in high concentrations, the importance of super-resolution methods, including structured illumination microscopy (SIM), photoactivated localization microscopy (PALM), stochastic optical reconstruction microscopy (STORM), and stimulated emission depletion microscopy (STED), is highlighted, because they can break through the diffraction limit (≈200 nm); thus, the boundary between one single molecule and another is no longer blurred. Since the imaging speed of PALM and STORM is slow, they are not suitable for high-speed single-molecule tracking (>1 μm^2^/s). Recently, PALM was successfully used to track slow-moving proteins in living roots [90]. It can be expected that the combined applications of TIRF-SIM [91] and STED-FCS [92] will be used in plant research in the near future. Recently, some other revolutionary technologies have emerged. By segmenting the back focal plane to image the same fluorophore from different angles, researchers found that single molecule light field microscopy (SMLFM) achieved 20 nm precision [93]. By taking advantage of a tilted light sheet and point spread functions, researchers built TILT3D (tilted light sheet microscopy with 3D point spread functions) [94], which can realize a resolution of tens of nanometers. Using a repetitive optical selective exposure technique, Tao Xu’s and Wei Ji’s groups realized ≈3 nm localization precision [95]. Stefan W. Hell’s group developed a localizing method called MINFLUX (minimal photon fluxes) to attain ≈1 nm spatiotemporal resolution in living cells by localizing individual switchable fluorophores using a donut-shaped excitation beam [96,97]. In addition, SR-CLEM (super-resolution correlative light and electron microscopy) is also worth investigating [98].

## 4. Applications in Living Plant Cells

### 4.1. The Advantages of Single-Molecule Techniques

Single-molecule imaging has the following three advantages compared with traditional imaging: first, because of its single-molecule sensitivity, it only requires a very small number of samples, which is beneficial for studying samples that are not easily detectable; second, it has nanometer-level spatial (especially combined with super resolution) and millisecond-level temporal resolution; and finally, since individual fluorescence-labeled biomolecules are detected separately, the molecular heterogeneities, which are lost by ensemble averaging in traditional biochemical and biophysical assays, will be truly obtained. Single-molecule imaging can obtain the single-molecule real-time trajectory and conformational changes of targeted molecules, from which the static details including location; distribution and polymerization; and dynamic details such as intermediates, interaction, and movement parameters can be directly captured. Taking advantage of the above, many unprecedented details about the behaviors and functions of specific types of molecules in complex biological processes have been revealed. Below, we provide examples of the single-molecule technique in plants.

### 4.2. Measurement of Protein Complexes

Protein exists in different forms in plant cells, and most protein molecules perform functions in the form of oligomeric proteins [99]. Conventional experiments, such as co-immunoprecipitation and fluorescence complementation, are difficult to directly detect the true oligomeric state and dynamics of biomolecules in living cells. Compared with traditional molecular experiments, single-molecule imaging can directly quantify the degree of aggregation in real time, which is of great significance for understanding the process of protein functioning. One protein is fused to one fluorophore; thus, the degree of polymerization can be detected from counting the number of photobleaching steps, or from the spot fluorescence intensity compared to the value of a single fluorophore under the same excitation [16]. Here, the importance of fluorescent proteins as monomers is further highlighted. Below, we list a few examples of single-molecule technology in revealing the form of protein aggregates to mediate life processes. As the time of blue light irradiation was prolonged, the ratio of phot1-GFP dimerization at the plasma membrane in *Arabidopsis* gradually increased compared to the ratio of monomers, indicating that blue light can induce phot1 dimerization [33]. After external high-ammonium treatment, trimers of AMT1;3 were found to aggregate into large clusters, which further internalized into the cytoplasm. This indicates that plants store active AMT1;3 on the cell membrane to avoid the absorption of ammonium ions [100]. AtHIR1 oligomerization was promoted by microdomains and the cytoskeleton in response to pathogens [17]. These examples illustrate the importance of real-time detection of protein aggregates to gain a deeper understanding plant life processes.

### 4.3. Quantification of Protein Dynamics and the Interaction of Single Molecules

Single-molecule fluorescence technology provides high location accuracy and temporal resolution. By recording the position of a single fluorescent molecule over time, its precise movement trajectory can be obtained in real time [101]. Then, the dynamic information of the target molecule can be extracted, and the dynamic parameters such as movement patterns and migration rate can be quantified. This application is suitable for studying how plants respond to external stimuli. For example, using single-molecule imaging, water transport protein PIP2;1 internalization was found to be significantly accelerated under salt treatment [16]. In addition to single-molecule technology being used to study the response of plants to stress signals, the transduction of plant hormone signals has also been involved. The diffusion coefficient of BRI1 protein increased significantly after brassinosteroid (BR) stimulation, indicating that BRs can significantly activate its receptor, leading to faster diffusion, which may be a necessary condition for further signal transduction [102]. The phosphorylation of NRT1.1 not only affected its oligomerization but also modulated its lateral mobility on the plasma membrane, and thereafter regulated auxin flux [19]. JA (jasmonic acid) signaling induced the endocytosis of AtRGS1 and its dissociation from AtGPA1 to activate heterotrimeric G proteins [84]. Detecting and elucidating protein interactions is crucial to understanding the biochemical mechanisms behind them. Using dual-color tracking, the co-localization degree of BRI1-GFP and AtFlot1-mCherry increased at high BR levels [102].

## 5. Future Prospects

Here, we have provided a methodological review of single-molecule imaging in plant cells. Single-molecule imaging is a powerful method that sheds new light on old problems, and its use is becoming more widespread. However, its applications lag, and there are some unique limitations (mainly due to thick cell walls and high-degree auto-fluorescence) in plants compared with those in animal cells. For labeling, the cell wall is an obstacle. Single-molecule imaging in protoplasts may be a viable alternative. The use of self-labeling protein tags is promising because there is a wide variety of fluorescent dyes with high fluorescence intensity and light stability, and one type of protein can be labeled by different fluorophores, which is helpful for multicolor imaging. For plants, washing off unlabeled fluorescent molecules is indeed a significant problem, but with the development of wash-free fluorescent dyes [103,104], this situation will gradually improve.

For imaging modalities, at present, co-localization has been used to study single-molecule protein interactions. However, sometimes co-localization may not reflect the real situation. In particular, two-dimensional co-localization easily causes errors. Single-molecule fluorescence resonance energy transfer (smFRET) is a powerful method in terms of studying biomacromolecule interactions in real time. A donor transfers its energy to an acceptor, and the energy transfer efficiency is inversely related to distance. Therefore, researchers can infer the distance between molecules by the energy transfer efficiency calculated from the fluorescence intensity of the donor and acceptor. Fluorescence lifetime imaging microscopy (FLIM) is based on using the differences in the natural lifetime (not wavelength and intensity) of fluorophores to detect and distinguish fluorescent molecules [105]. This technique provides another option for fluorescent molecules that cannot be distinguished by spectrum and can remove the interference of autofluorescence. It can also be used for molecule tracking [106]. In addition, FRET–FLIM has been used to study protein interactions in plant roots [107]. Although smFRET/smFRET–FLIM has not yet been implemented in plants, it is just around the corner.

Although the most widely used single-molecule imaging method in plants is TIRF or HILO, there is no doubt that LSFM with a wide range of spatial resolution (from the individual to the single-molecule level) is the most promising imaging method for future applications. From the perspective of imaging principles, HILO can be considered the first generation of light sheet imaging. The signal-to-noise ratio of LSFM is close to that of TIRF, and its signal-to-noise ratio will be further improved if thinner light sheets are used. More importantly, it bypasses the main limitation of TIRF, which can only image near-membrane. In addition, the application of LSFM–SIM in plants is promising [108].

To date, single-molecule imaging is the only way to faithfully monitor the location and dynamics of individual biomolecules with spatial and temporal heterogeneities in situ. Many single-molecule labeling and imaging methods have been developed in animals, and labeling and imaging technique developments are still a research hot spot. However, the application of these technologies in plants and their optimization according to the specific properties of plants still require in-depth studies, although the basic principles of imaging are the same. Although this field is relatively narrow and young, some successful results that further our understanding of the basic biological signals of plants have been achieved. These promising results are the reason for optimism to believe that with the joint efforts of physicists, chemists, and biologists around the world, all plant science-related mechanisms will be explained at the single-molecule level in the future.

## Data Availability

Not applicable.

## Abbreviations

3D-SMART	3D single-molecule active real-time tracking method
BR	brassinosteroid
CDots	carbon-based nanodots
FCS	fluorescence correlation spectroscopy
FCCS	fluorescence cross-correlation spectroscopy
FLIM	fluorescence lifetime imaging microscopy
FNDs	fluorescent nanodiamonds
GFP	green fluorescent protein
HILO	highly inclined and laminated optical sheet
JA	jasmonic acid
LSFM	light sheet fluorescence microscopy
MINFLUX	minimal photon fluxes
PALM	photoactivated localization microscopy
PDots	polymer dots
QDs	quantum dots
smFRET	single-molecule fluorescence resonance energy transfer
SIM	structured illumination microscopy
SMLFM	single molecule light field microscopy
SR-CLEM	super-resolution correlative light and electron microscopy
STED	stimulated emission depletion microscopy
STORM	stochastic optical reconstruction microscopy
TILT3D	tilted light sheet microscopy with 3D point spread functions
TIRF	total internal reflection fluorescence
TIRFM	total internal reflection fluorescence microscopy
UCNPs	up-conversion nanocrystals
